# Early breast cancer screening using iron/iron oxide-based nanoplatforms with sub-femtomolar limits of detection

**DOI:** 10.3762/bjnano.7.33

**Published:** 2016-03-07

**Authors:** Dinusha N Udukala, Hongwang Wang, Sebastian O Wendel, Aruni P Malalasekera, Thilani N Samarakoon, Asanka S Yapa, Gayani Abayaweera, Matthew T Basel, Pamela Maynez, Raquel Ortega, Yubisela Toledo, Leonie Bossmann, Colette Robinson, Katharine E Janik, Olga B Koper, Ping Li, Massoud Motamedi, Daniel A Higgins, Gary Gadbury, Gaohong Zhu, Deryl L Troyer, Stefan H Bossmann

**Affiliations:** 1 Kansas State University, Department of Chemistry, 213 CBC Building, Manhattan, KS, USA; 2 Kansas State University, Department of Anatomy & Physiology, 228 Coles Hall, Manhattan, KS, USA; 3 The University of Texas Medical Branch, 301 University Boulevard, Galveston, TX, USA; 4 Kansas State University, Department of Statistics, 101 Dickens Hall, Manhattan, KS, USA; 5 The First Affiliated Hospital of Kunming Medical University, Department of Nuclear Medicine, 295 Xichang Road, Kunming, Yunnan, PR China

**Keywords:** biophotonics, breast cancer, iron/iron oxide nanoparticle, liquid biopsy, nanodiagnostics detection, nanomedicine, sub-femtomolar limit of detection

## Abstract

Proteases, including matrix metalloproteinases (MMPs), tissue serine proteases, and cathepsins (CTS) exhibit numerous functions in tumor biology. Solid tumors are characterized by changes in protease expression levels by tumor and surrounding tissue. Therefore, monitoring protease levels in tissue samples and liquid biopsies is a vital strategy for early cancer detection. Water-dispersable Fe/Fe_3_O_4_-core/shell based nanoplatforms for protease detection are capable of detecting protease activity down to sub-femtomolar limits of detection. They feature one dye (tetrakis(carboxyphenyl)porphyrin (TCPP)) that is tethered to the central nanoparticle by means of a protease-cleavable consensus sequence and a second dye (Cy 5.5) that is directly linked. Based on the protease activities of urokinase plasminogen activator (uPA), MMPs 1, 2, 3, 7, 9, and 13, as well as CTS B and L, human breast cancer can be detected at stage I by means of a simple serum test. By monitoring CTS B and L stage 0 detection may be achieved. This initial study, comprised of 46 breast cancer patients and 20 apparently healthy human subjects, demonstrates the feasibility of protease-activity-based liquid biopsies for early cancer diagnosis.

## Introduction

We have detected stage I breast cancer in human patients with statistical significance by means of a simple serum test using highly sensitive Fe/Fe_3_O_4_-nanoparticle based nanoplatforms for protease detection. Numerous proteases are required for early mutations, tumor survival, progression, angiogenesis, and invasion [1–3]. Following the pioneering research of Weissleder et al. [4], molecular [5], macromolecular [6] and nanoparticle-based [7] protease sensors have been developed for in vivo imaging and in vitro diagnostics of proteases that rely on fluorescence and magnetic principles [8]. This technology is characterized by high versatility and specificity, because consensus sequences feature high selectivities for the proteases for which they were designed [9]. However, the limits of protease detection (LOD’s) of the state-of-the-art technology are sub-picomolar (sub-ng/mg) [4–8], which is sufficient for in vivo imaging of tumors [4,8], atherosclerotic plaques [10] and cardiovascular inflammation [11] in humans and in vivo and in vitro detection in rodent models for cancers [12–13], but not for the in vitro detection of human cancers [14] in their earliest stages. Competing technologies for quantitative protease detection, such as immunosorbent assays [15], quantum dot barcode technology [16], and immunobeads [17] have similar LOD’s. Recently, Sardar, Korc et al. have reported the sensing of short noncoding RNA following a nanoplasmonic approach, which is of similar sensitivity and range as the approach reported here [18].

**Figure 1:**
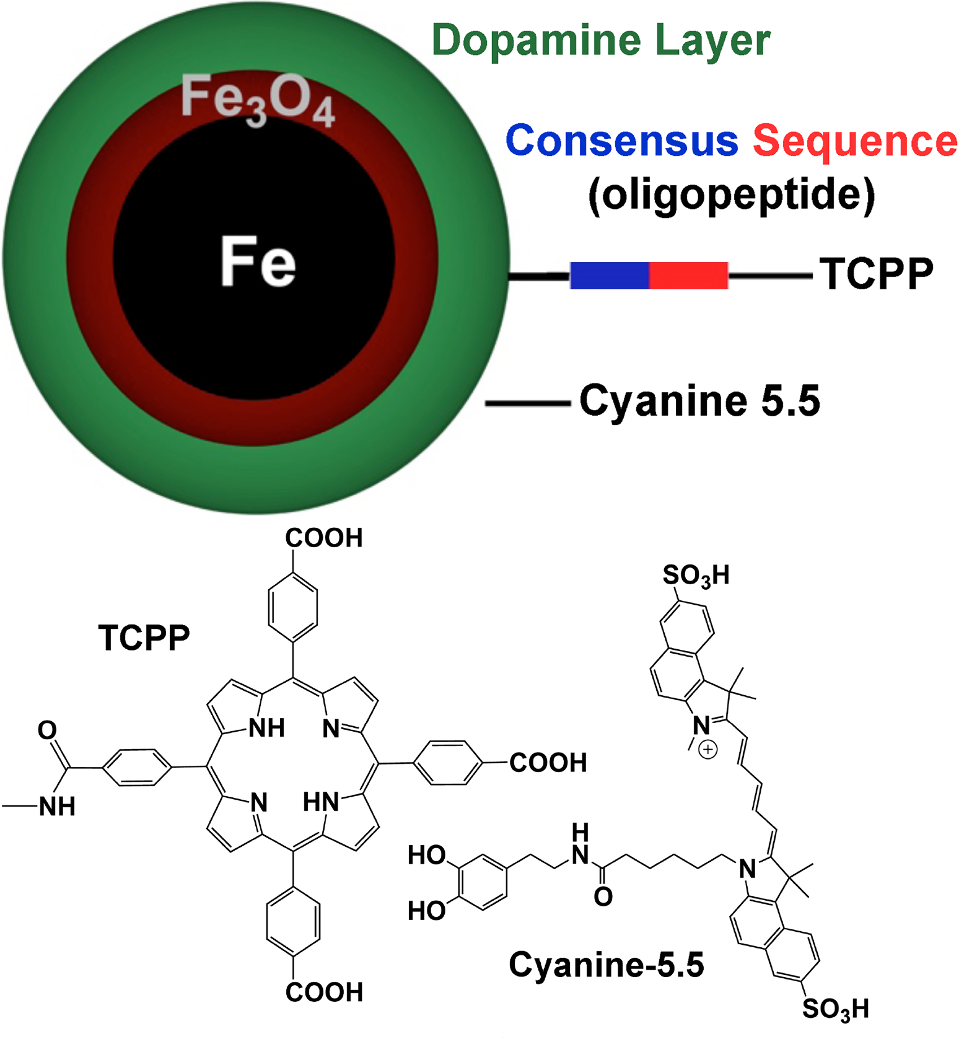
Nanosensors for in vitro protease detection. For each protease, a highly selective oligopeptide is used to tether tetrakis-carboxy-phenyl-porphyrin (TCPP) to the nanoparticle. Cyanine 5.5 is linked permanently to the Fe/Fe_3_O_4_ nanoparticles. Figure 1 is reproduced from [23] with permission.

We have developed nanoplatforms for protease detection [19–20] that are capable of detecting protease activities over a wide activity range down to sub-femtomolar LOD’s. These nanoplatforms consist of dopamine-covered, water-dispersable iron/iron oxide core/shell nanoparticles, to which one fluorescent dye (TCPP, tetrakis(carboxyphenyl)porphyrin) is tethered via a consensus sequence. A second dye (cyanine 5.5) is permanently linked to the dopamine coating (Figure 1). This design enables both, plasmon-resonance quenching (SET) [20–21] and Förster resonance energy transfer (FRET) quenching [20,22] of the tethered TCPP units. Once TCPP is released via proteolytic cleavage of the consensus sequence, its fluorescence will increase (for most of the nanoplatforms).

**Figure 2:**
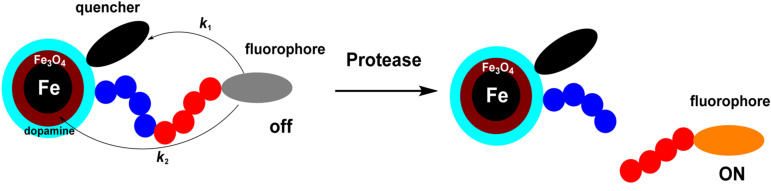
Mechanistic scheme of the “light switch effect” upon proteolytic cleavage: the fluorophore is switched on due to the increase in distance between the Fe/Fe_3_O_4_ core/shell nanoparticle, leading to decreased Förster resonance energy transfer (FRET) [21,24], *k*_1_, and dipole–surface energy transfer (SET) [20,22], *k*_2_. Further explanations are provided in the text.

The nanoplatforms for cancer detection are based on proteolytic cleavage of TCPP from the Fe/Fe_3_O_4_-core (Figure 2). Increasing the distance between the TCPP fluorophore and the nanoparticle decreases plasmon-resonance quenching (dipole–surface energy transfer (SET) [20–21]) from TCPP to Fe/Fe_3_O_4_ and Förster resonance energy transfer (FRET [20,22]) from TCPP to cyanine 5.5. The latter is permanently tethered to the inorganic nanoparticle. For all of the employed consensus sequences, with the exceptions of GAGSGR-SAG for uPA and GAGVPLS-LYSGAG for MMP 9, an increase in TCPP fluorescence is observed upon enzymatic cleavage. This “light switch effect” [20] enables highly sensitive detection of protease activity by quantitative fluorescence measurements. In an earlier paper, we have discussed in detail why the nanoplatforms for uPA and MMP 9 detection defy the general paradigm: shorter consensus sequences and sequences permitting higher dynamics of the attached TCPP lead to fluorescence enhancement of the attached fluorophore due to enhanced plasmonic light scattering [24] of the Fe(0) core of the central core/shell nanoparticle. For these specific consensus sequences, this effect exceeds the quenching effects (SET and FRET). Therefore, these two nanoplatforms show decreases of TCPP fluorescence upon cleavage. However, this decrease can still be utilized to measure the activities of uPA and MMP 9 in serum.

In the US, breast cancer is staged according to the TNM classification system, which is based on the extent of the spread of cancer within the body [25]. The overall 5-year survival rates for breast cancer are virtually 100% at stages 0 and I, 93% at stage II, 72% at stage III and 22% at stage IV [26]. 61% of all breast cancers in the US are diagnosed at combined stages 0 and I, 32% at stage II and 7% at combined stages III and IV [27]. Since the majority of breast cancer mortalities occurs from cases that are detected at stages II and above, detecting breast cancer by means of a routine blood test at stage I or earlier would have the potential of significantly reducing breast cancer mortality (521,900 globally in 2012) [28].

Bhatia et al. proposed nanoscale agents for in vivo use that are comprised of reporter molecules bound via consensus sequences to iron oxide nanoworms. The reporter molecules are released in rodent models once the nanoworms have reached the cancer site and then excreted in urine. The quantitative detection of the reporter molecules’ concentrations has been achieved by paper chromatography [29]. Although this was a major step forward in developing point-of-care diagnostics, it is still more than minimally invasive, because the nanoworms have to be given intravenously. An ideal “liquid biopsy” [30] will require only the drawing of a simple blood sample to detect cancer, without introducing a reagent to the patient’s body first. In this report, we would like to discuss this approach.

**Figure 3:**
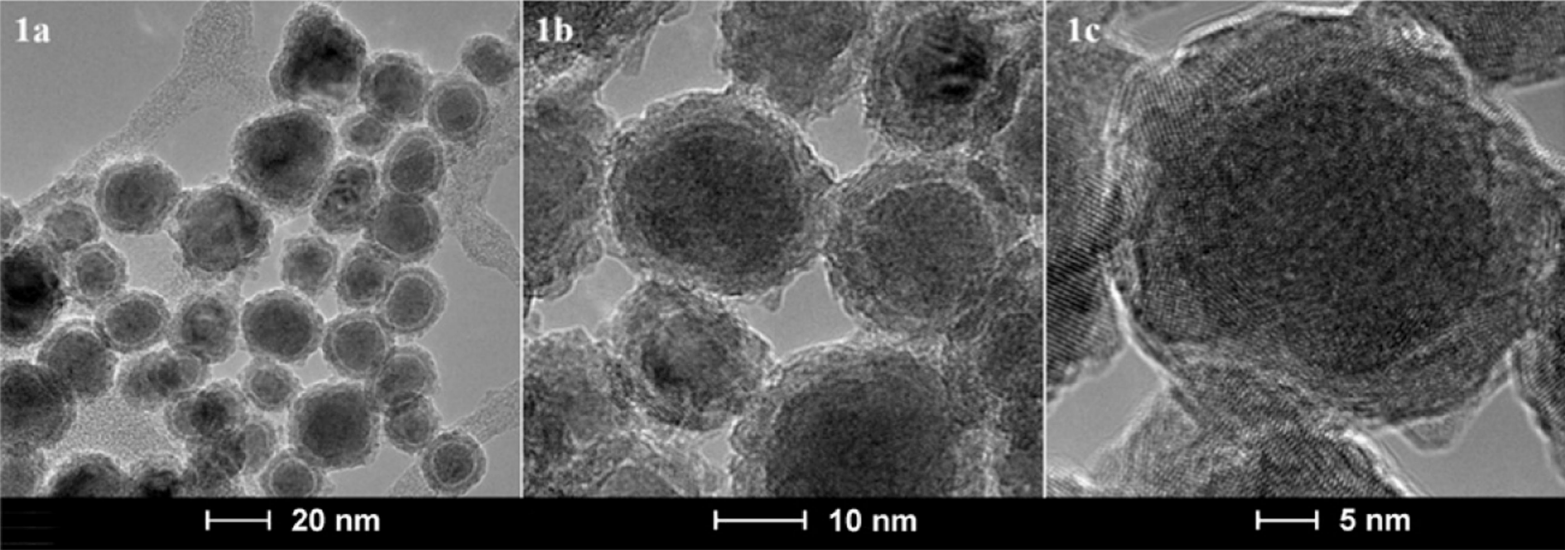
TEM (1a,1b) and HRTEM (1c) images of Fe/Fe_3_O_4_-core/shell nanoparticles that are forming the inorganic core of the nanoplatforms for protease detection, with permission from [20], copyright 2014 Royal Society of Chemistry. HRTEM images revealed that the Fe(0) centers are mostly crystalline (BCC).

In 2014, we published the synthesis and calibration of Fe/Fe_3_O_4_-based nanoplatforms for accurate and highly sensitive detection of 12 proteases (Figure 1) [20]. The calibration and validation experiments were performed with commercially available proteases in PBS (phosphate buffered saline, pH 7.4). The average Fe(0) core diameter is 13 ± 0.5 nm, the Fe_3_O_4_ shell thickness is 2.0 ± 0.5 nm (Figure 3). Using statistical modeling, the optimal number of TCPP units per nanoparticle was determined to be 35 ± 3, and the number of cyanine 5.5 units to be 50 ± 4 [31].

We have obtained serum samples (−80 °C) from 46 female breast cancer patients (4 stage 0, 9 stage I, 9 stage II, 12 stage III and 12 stage IV, as well as 20 healthy human subjects (10 males and 10 females)) from the Southeastern Nebraska Cancer Center. We have selected serum as biospecimen, because at −80 °C protease activity is retained for years according to our preliminary results. 20 breast cancers were luminal A [32], 12 were luminal B [33], 8 were basal-like [32] and 6 were HER2 enriched [32]. All patients (ages 36 to 80) and healthy human subjects (ages 26 to 68) were Caucasian. No significant statistical differences in the protease expression pattern between the females and males of the control group were found.

Approx. two percent of the human genome encodes for proteases [34]. Therefore, each selection of proteases for a cancer diagnostic panel is somewhat arbitrary. For detecting early breast cancer, we have chosen the following proteases: MMPs 1, 2, 3, 7, 9, 13, uPA and CTS B and L. MMP 1 has been associated with telomerase activity and promotion of tumor invasiveness and metastatic dissemination [35]. MMPs 2, 7, and 9, as well as other MMPs, release growth factors from stromal and epithelial cells at the cancer boundary, cleave off pro-angiogenic factors and start pro-angiogenic protease cascades [36–37]. MMP 13 is involved in the epithelial-mesenchymal transition [38]. uPA and CTS B facilitate angiogenesis, ECM degradation and invasiveness. They also activate growth factors [39–40]. MMP3 and CTS L are responsible for early mutations in carcinogenesis [2–3].

## Results and Discussion

In step 1, the influence of the serum matrix on the performance of the nanoplatforms was evaluated. For this purpose, we have used combined serum from our control group, which was inactivated using established procedures by heating to 56 °C for >30 min [41].

In short, 3.0 mL of dextran (10 mg dextran in 1.0 mL of PBS) were mixed with 75 µL of the nanoplatform dispersion (1.0 mg in 1.0 mL of PBS) and 30 µL of the protease stock solutions at each concentration level in a total volume of 3.0 mL of PBS. 30 µL of inactivated serum was added before filling up to 3.0 mL when studying matrix effects. The solution was incubated at 25 °C for 60 min. Then the fluorescence was analyzed in 4.0 mL quartz-cuvettes (Helma) using a spectrofluorometer (Fluoromax2) with dual monochromators (λ_ex_ = 421 nm, λ_em_ = 620–680 nm). The complete procedure is described in the Methods section. From 10 independently performed repetitions, we have calculated the experimental error to ±3% (Supporting Information File 1, Figure S3).

**Figure 4:**
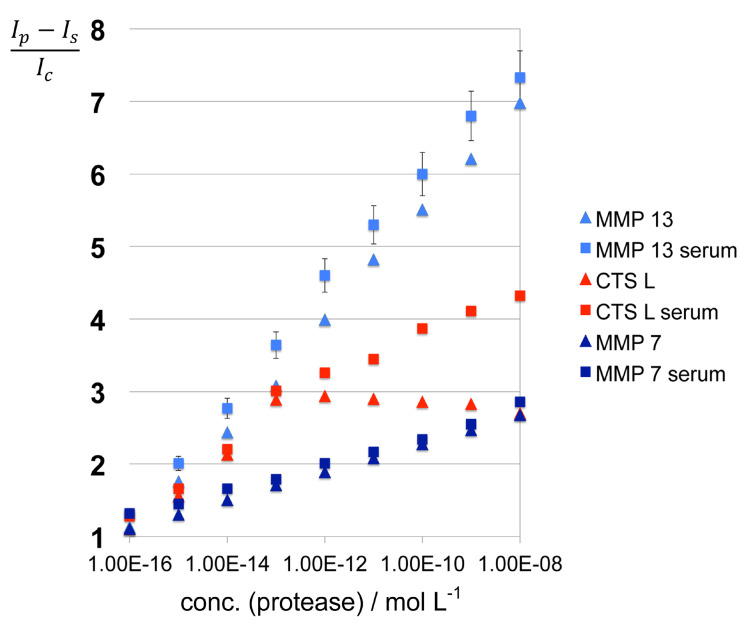
Matrix effects for MMP7, MMP13, and cathepsin L after 60 min of incubation at 25 °C under standard conditions. *I_p_*: fluorescence signal after 60 min of incubation; *I_c_*: fluorescence signal in the absence of protease after 60 min incubation; *I_s_*: fluorescence signal of serum/PBS-dextran alone. Experimental errors are indicated.

**Figure 5:**
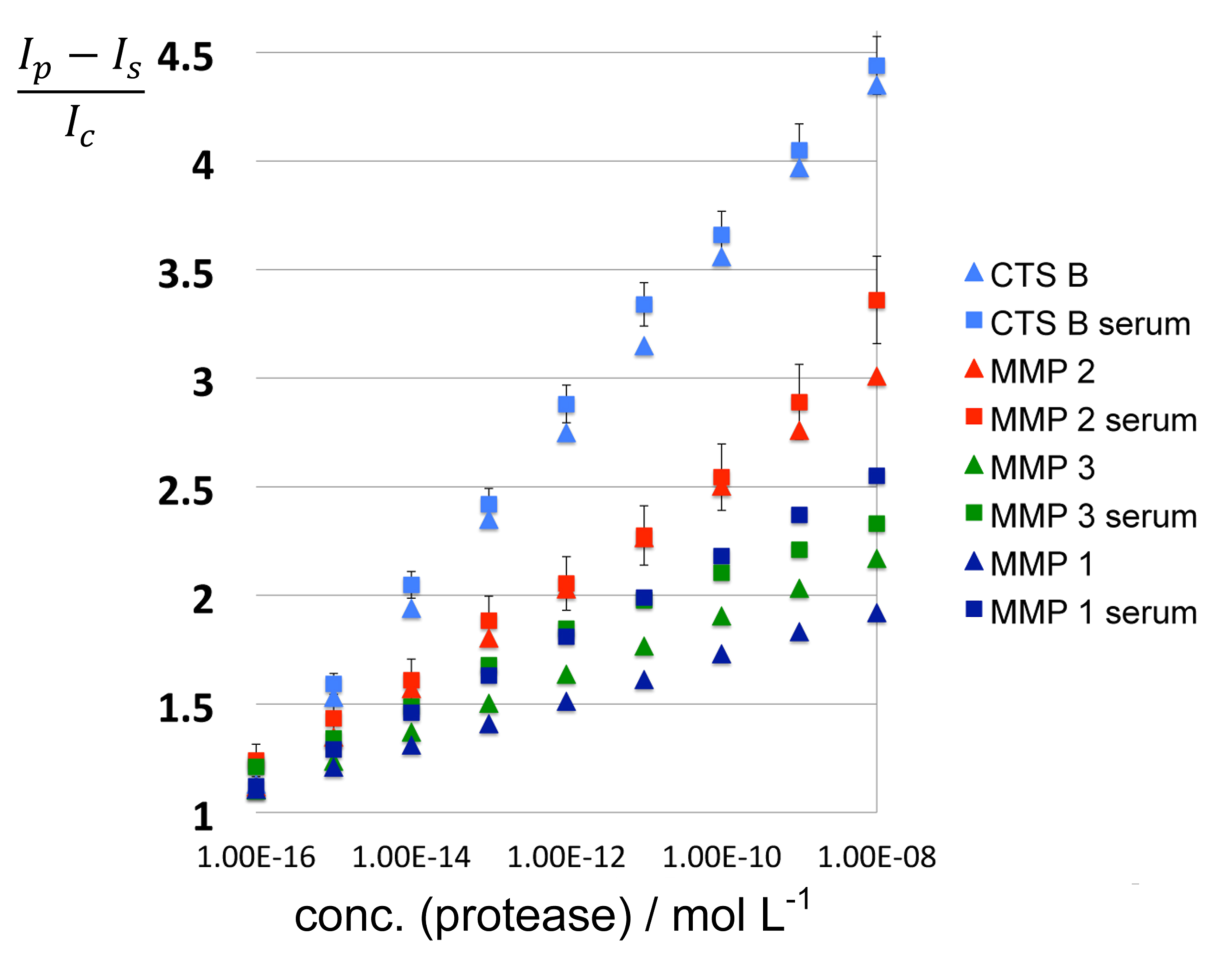
Matrix effects for MMP1, MMP 2, MMP 3, and cathepsin B after 60 min of incubation at 25 °C under standard conditions. Triangles: fluorescence readings in PBS; Squares: fluorescence readings in PBS containing inactivated serum. *I_p_*: fluorescence signal after 60 min of incubation; *I_c_*: fluorescence signal in the absence of protease after 60 min incubation; *I_s_*: fluorescence signal of serum/PBS-dextran alone. Experimental errors are indicated.

The results obtained in the presence and absence of inactivated serum are shown in Figure 4 and Figure 5, as well as Figure S1 in Supporting Information File 1 (Triangles: fluorescence readings in PBS; Squares: fluorescence readings in PBS containing inactivated serum.) Most proteases only exhibit moderate matrix effects, because of the very low concentration of serum that is required and due to the use of dextran as anticoagulant [42]. The requirement of only a very low volume of serum for performing meaningful enzyme activity measurements is a definite advantage of the very high sensitivity of the nanoplatforms for protease detection, which originates from the concurrent utilization of SEM and FRET quenching.

Notable exceptions are MMPs 1 (Figure 5) and 7 (Figure 4) where significant matrix effects were detected. As noted in Table S1 in Supporting Information File 1, the physical properties (isoelectric point and hydrophobicity index) of the consensus sequences plus peptide linkers designed for detecting MMP 1 and MMP 7 are within the ranges defined by all employed peptide sequences. Pieper et al. have analyzed human serum by fractionating serum proteins, followed by two-dimensional electrophoresis, and sequential anion-exchange and size-exclusion chromatography. They have resolved 3700 posttranslationally modified proteins [43]. Based on their findings, we cannot exclude that binding of the peptide sequences designed for MMP 1 and MMP 7 detection to one or several serum protein occurs, which is ultimately responsible for the observed photophysical behavior of these nanoplatforms.

In Supporting Information File 1, Figure S1 the results for uPA and MMP9, two proteases that defy the “light switch paradigm” are shown. An explanation for this behavior is briefly discussed in the Introduction section and more thoroughly in [20].

### Cross-sensitivities of the nanoplatforms

**Figure 6:**
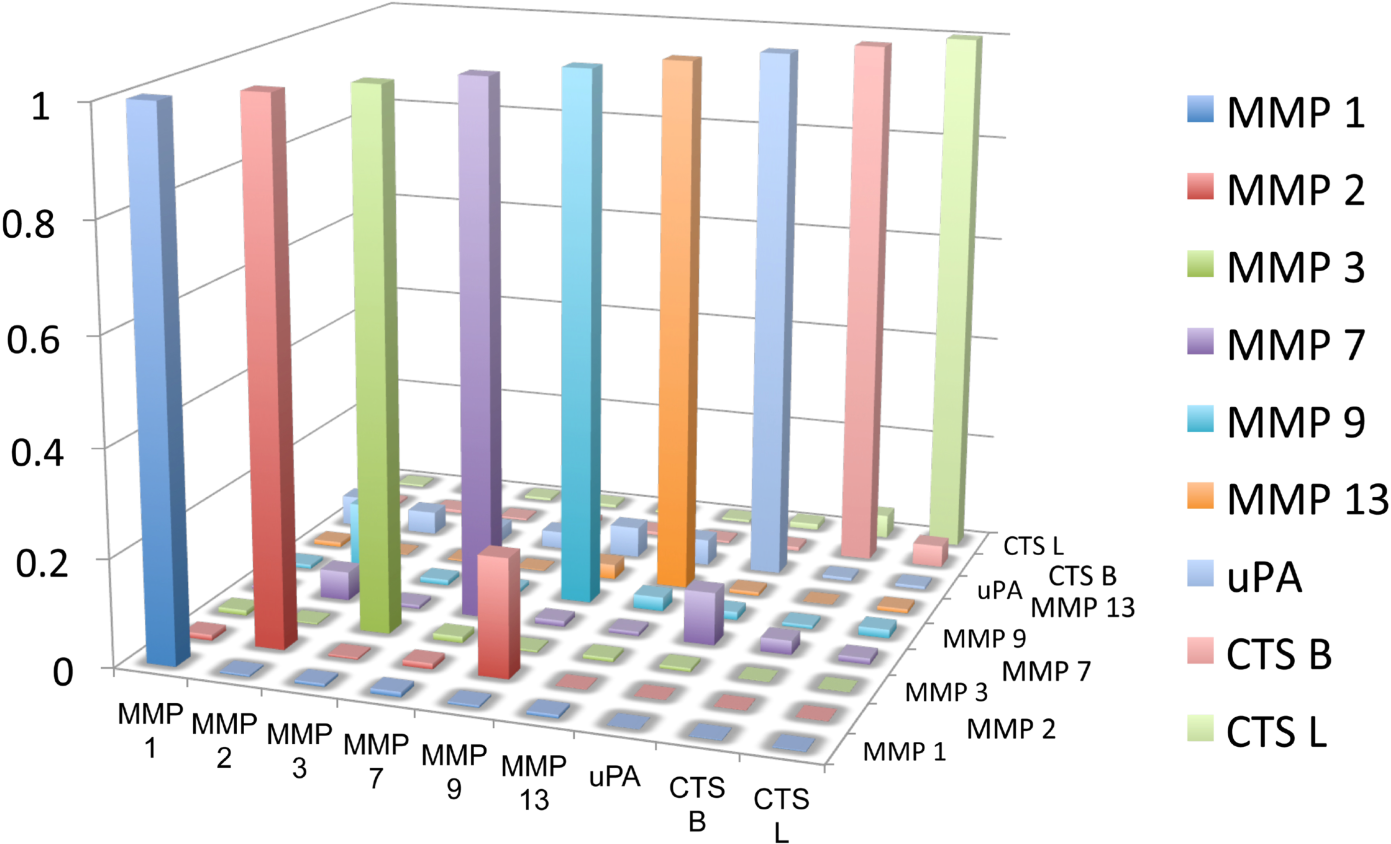
Cross-sensitivities of the nanoplatforms used in this study. Further explanations are provided above.

In order to determine the cross-sensitivities of the nanoplatforms, the following control experiments were conducted: The nanoplatforms for MMP 1, 2, 3, 7, 9, 13, uPA, and CTS B, L were (separately) incubated with 1.0 × 10^−10^ mol L^−1^ of MMP 1 under standard conditions (see Methods). After 60 min of incubation at 25 °C, the fluorescence spectra of all nanoplatforms were recorded. The next set of experiments consisted of incubating the nanoplatforms for MMP 1, 2, 3, 7, 9, 13, uPA, and CTS B, L with 1.0 × 10^−10^ mol L^−1^ of MMP 2 under standard conditions. This is followed by MMP 3, 7, 9, 19, uPA and CTS B, and L. In Figure 6, the normalized results for this set of experiments are summarized. The normalization procedure consists of dividing each set of integrated fluorescence data for each enzyme by the fluorescence recording for the correct match in the entire set of nine nanoplatforms.

Set 1: integrated fluorescence recordings for all nine nanoplatforms incubated with MMP 1 (1.0 × 10^−10^ mol L^−1^), divided by the integrated fluorescence signal obtained with the nanoplatform for MMP 1 in the presence of MMP 1.

Set 2: integrated fluorescence recordings for all nine nanoplatforms incubated with MMP 2 (1.0 × 10^−10^ mol L^−1^), divided by the integrated fluorescence signal obtained with the nanoplatform for MMP 2 in the presence of MMP 2.

Sets 3 to 8 have been recorded accordingly for MMP 3, 7, 9, 13, uPA and CTS B.

Set 9: integrated fluorescence recordings for all nine nanoplatforms incubated with CTS L (1.0 × 10^−10^ mol L^−1^), divided by the integrated fluorescence signal obtained with the nanoplatform for CTS L in the presence of CTS L.

### Diagnosis of early breast cancer

**Figure 7:**
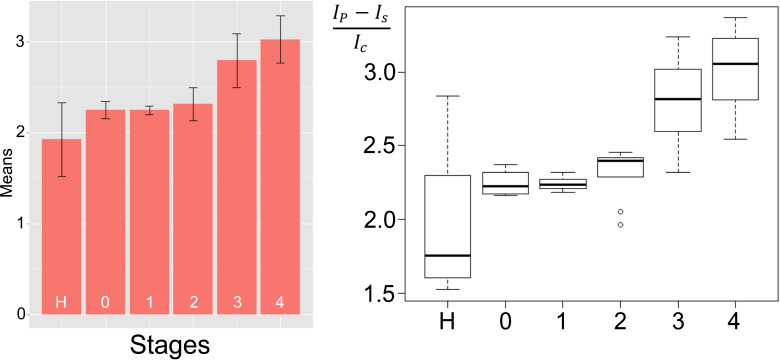
Bar graph (left, showing means and standard deviations) and box plot (right, indicating the observed data range) for cathepsin L. The group sizes are H (apparently healthy control group, *n* = 20), 0: breast cancer stage 0 (*n* = 4), 1: breast cancer stage 1 (*n* = 9), 2: breast cancer stage 2 (*n* = 9), 3: breast cancer stage 3 (*n* = 12); 4: breast cancer stage 4 (*n* = 12). All biospecimens were obtained from the South Eastern Nebraska Cancer Center (SNCC). Breast cancer has been staged according to the TNM staging system [25].

The activities of the nine selected proteases in the serum of 46 breast cancer patients and 20 healthy human subjects were measured following the same procedure as for determining the matrix influence, with the exception that active serum was used, and the results statistically analyzed. A series of boxplots and bar graphs (Figure 7 and Supporting Information File 1, Figures S4–S12) show the data range that correlates to each cancer stage, as well as the protease expression range of healthy patients [44].

The analyzed enzymes include cathepsin B and L, MMP 1, 2, 3, 7, 9, 13 and uPA. Except for MMP9 and uPA, all enzymes display a positive trend with an increasing signal for higher cancer stages. The reason for this behavior is discussed in the text: in short, the nanoplatforms for uPA and MMP 9 detection show decreasing fluorescence intensities with increasing protease activity. We have chosen boxplots and bar graphs for data analysis, in combination with Welch two sample t-tests (control group and cancer patients at a defined stage) [45], because a combination of these analysis methods provides a simple system for data analysis. The boxplots show the data range that correlates to a certain cancer stage while the bar graphs display the average signal and standard deviation (represented by the error bar) for individual cancer stages.

With respect to detecting cancer at an early stage, the data obtained for cathepsin B and L, uPA and MMP 1, 3 and 9 is superior to MMP 2, 7 and 13. Here the fluorescence signals for each cancer stage are compared with the healthy control group’s fluorescence signals. Highly significant differences between cancer patients and healthy control group are achieved with cathepsin B and L, uPA, MMP1 and 9. It is noteworthy that only cathepsins B and L are significantly different from the healthy group for stage 0 breast cancer. Especially cathepsin L seems promising here since it maintains its positive trend of the signal. However, the stage 0 group is very small (*n* = 4). Therefore, all enzymes should be revisited when more data becomes available.

Highly significant differences are achieved with CTS B, L, uPA, MMP 1 and 9. It is interesting to observe that only CTS B and L produce a signal for stage 0 breast cancer that is significantly different from the healthy group. Especially CTS L seems promising here since it maintains its positive trend of the signal. However, the stage 0 group is too small (*n* = 4) and the control group is somewhat spread out. Stage 0 must be revisited when more data becomes available.

**Figure 8:**
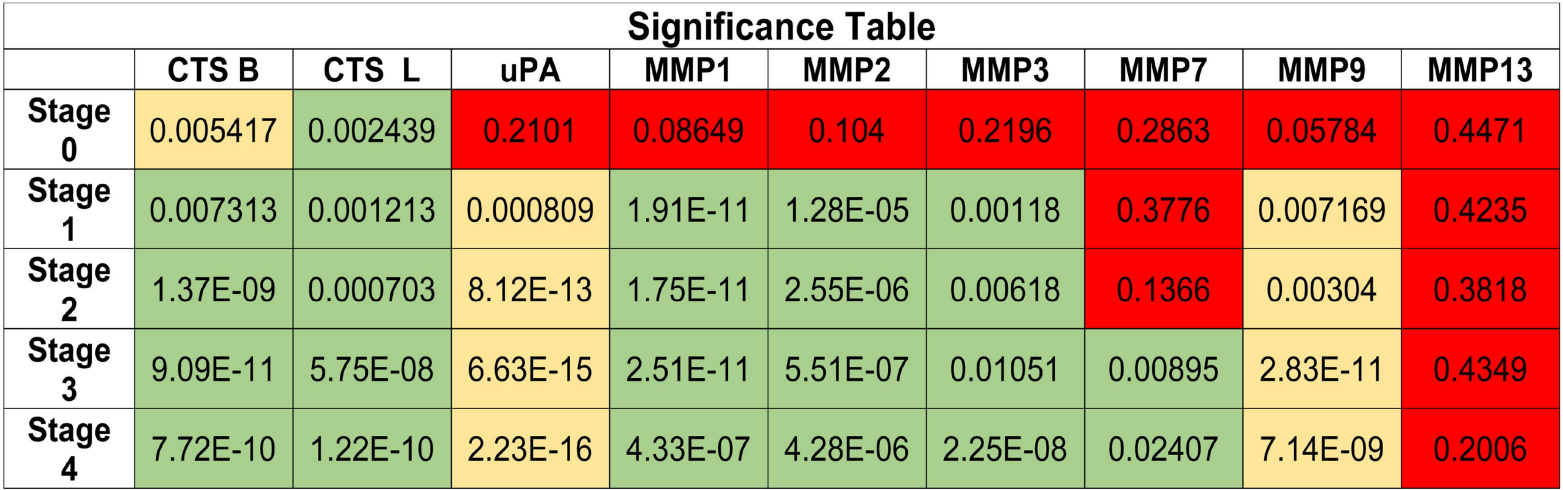
Calculated *p*-values; comparison of breast cancer patients and healthy human subjects for all investigated enzymes, stages 0–4. Green: fluorescence signal (FS) of cancer patients is significantly larger than of control group (CG); Yellow: FS is significantly smaller; Red: differences in FS and CG are not significant.

In Figure 8, the calculated *p*-values [45] obtained for comparisons of the protease expression pattern in each cancer stage with those of the healthy control group are tabulated, leading to the “Significance Table”. The color green denotes for measured fluorescence signals that are significantly enhanced (*p* < 0.05) in cancer patients compared to the healthy control group. The color yellow represents findings, in which the fluorescence signals detected in the serum of cancer patients were significantly smaller than in the control group. The color red was used for all cases in which significant results could not be obtained. It should be noted (again) that uPA and MMP 9 are the “defiant proteases”. Their fluorescence signals decrease with increased protease activity.

**Figure 9:**
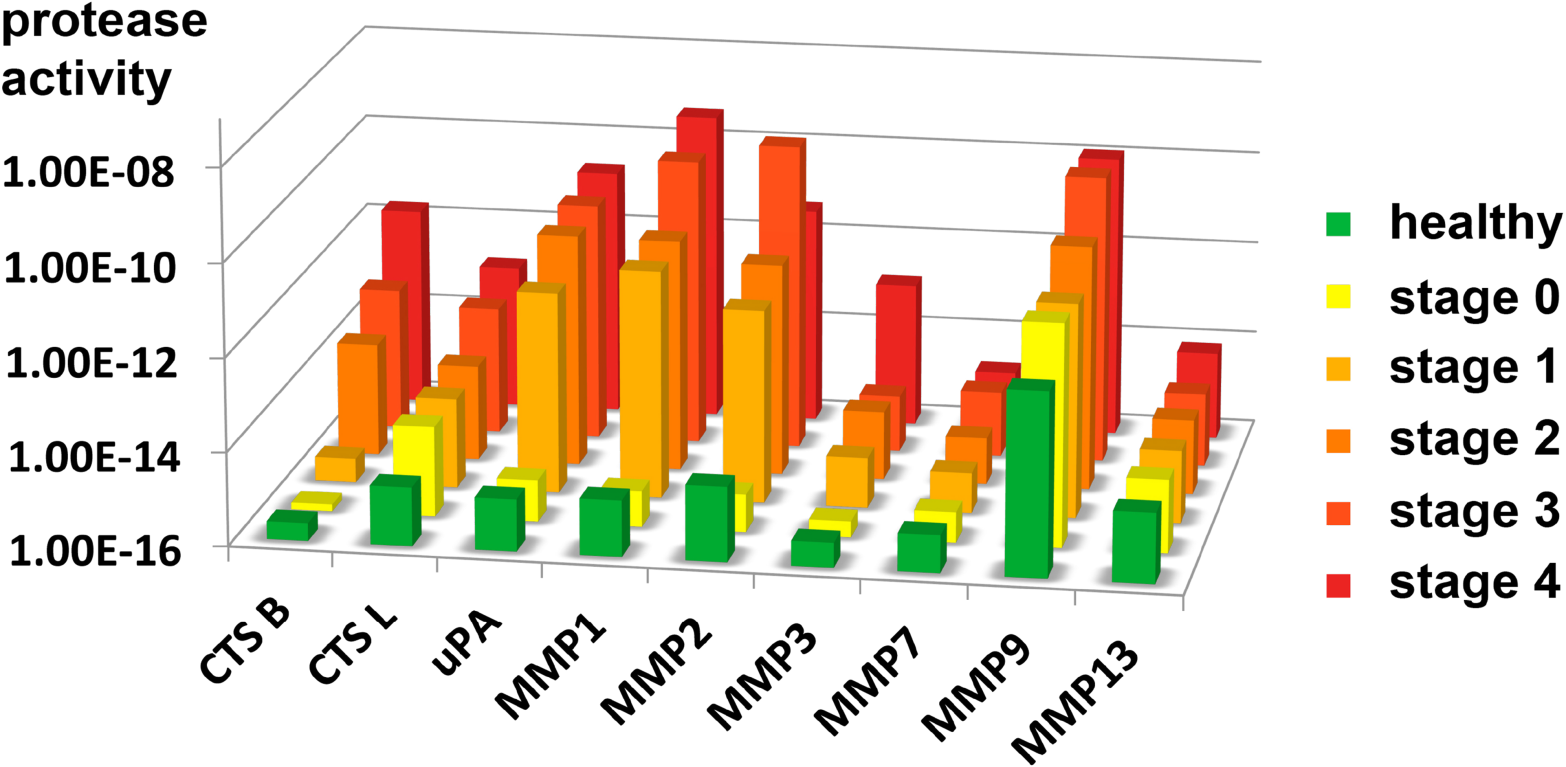
Average protease activity as a function of breast cancer stage/healthy control group for all nine proteases monitored in this study. Note that the activity is shown on a logarithmic scale (log_10_ (protease activity)). The data summarized in this figure is also reported in Supporting Information File 1, Tables S1–S9.

The resulting average enzyme activities in the serum of the healthy control group and breast cancer stages 0, 1, 2, 3, and 4 are summarized in Figure 9. Healthy control groups and stages are color coded. From this plot, it can be discerned why cathepsin L is the best enzyme to detect both, early breast cancer and cancer staging. MMP 1, MMP 9 and uPA show similar enzyme activity trends, but we were unable to distinguish between healthy patients and stage 0 breast cancer patients. The inability to reach this goal was due to variations of protease expression among the apparently healthy human subjects and the small sample size (*n* = 4) in stage 0. Cathepsin B, MMP 2 can be used to identify breast cancer patients that are in or beyond stage 2. MMP 3 could, in theory, identify late stage patients. Finally, MMP 7 and MMP 13 did not yield conclusive results. It is noteworthy that although MMP 2, 7, and 9 belong to a group of MMPs that are known to release growth factors, cleave off pro-angiogenic factors and start pro-angiogenic protease cascades [36–37], MMP 2 and MMP 9 yield conclusive results for stages one to four, whereas MMP 7 is only conclusive at higher stages. MMP 13 did not generate any significant results, although MMP 13 is involved in the epithelial-mesenchymal transition [38]. The reasons for these deviations among related matrix metalloproteinases may be found in different tissue retention and enzymatic degradation of individual proteases, as well as in the activity profiles of tissue inhibitors of metalloproteinases (TIMPs) in blood [46].

In conclusion, the most important result of this research is that we are able to detect breast cancer at stage I monitoring seven proteases and at stage 0 observing one protease with high statistical significance. This result is of importance, because we have achieved it with relatively small group sizes of breast cancer patients and healthy control subjects. As always when testing biomarkers, the selection process of the required biospecimens is crucial. Therefore, our next steps will consist in testing our liquid biopsy approach with significantly larger group sizes of stage 0 and I breast cancer patients.

## Methods

### Nanoplatform synthesis

The synthesis and characterization of the nanoplatforms for protease detection is described in detail in [19]. In short, water-dispersible Fe/Fe_3_O_4_ nanoparticles featuring dopamine ligands [47], TCPP [48], and cyanine 5.5 [49] were synthesized according to established procedures. The oligopeptides used as consensus sequences, which were synthesized in the Bossmann group by means of solid-supported peptide synthesis [20], are summarized in Table 1. TCPP was connected to the N-terminal end of the oligopeptides while it was still on the resin. The TCPP-oligopeptide was then cleaved off the resin and linked to the primary amine groups of Fe/Fe_3_O_4_ bound via an amide bond [20]. Note that these sequences also contain GAG and AG as peptide linkers.

**Table 1:** Consensus sequences in single-letter code for 9 proteases (http://www.lifetein.com/peptide-analysis-tool.html). Essential amino acids of the consensus sequences are bold.

Protease	Consensus sequence	Isoelectric point (pI)	Hydrophobicity index at pH 6.8

MMP1	GAG**VPMS**-**MRGG**AG	11.18	18.54
MMP2	GAG**IPVS**-**LRSG**AG	11.18	22.08
MMP3	GAG**RPFS**-**MIMG**AG	11.18	27.77
MMP7	GAG**VPLS**-**LTMG**AG	6.09	30.31
MMP9	GAG**VPLS**-**LYSG**AG	6.0	28.08
MMP13	GAG**PQGLA**-**GQRGIV**AG	11.18	19.88
uPA	GAG**SGR**-**SA**G	11.18	22.08
Cathepsin (CTS) B	GAG**SLLKSR**-**MVPNFN**AG	11.6	20.82
Cathepsin (CTS) L	GAG**SGVVIA**-**TVIVIT**AG	6.09	43.82

### Standard procedure of preparing protease assays (without serum)

3.0 mg of nanoplatform were dissolved in 3.0 mL of PBS. The dispersion was sonicated for 10 min. The resulting dispersion is chemically stable for 14 days at 4 °C. 900 mg of dextran were dissolved in 90 mL of PBS. Stock solutions of all 9 enzymes were prepared by consecutive dilution of commercially available proteases (Enzo Lifesciences). 3 mL of PBS–dextran (10 mg dextran in 1.0 mL of PBS) are mixed with 75 µL of the nanoplatform dispersion (3.0 mg in 3.0 mL of PBS, see above) and 30 µL of each of the proteases at every concentration level in PBS. The dispersions were incubated at 25 °C for 60 min, followed by the recording of a fluorescence spectrum at 25 °C using a Fluoromax2 spectrometer (λ_em_ = 421 nm, λ_ex_ = 620–680 nm).

### Standard procedure of preparing protease assays (with inactivated serum)

3.0 mg of nanoplatform were dissolved in 3.0 mL of PBS. The dispersion was sonicated for 10 min. The resulting dispersion is chemically stable for 14 days at 277 K. 900 mg of dextran were dissolved in 90 mL of PBS. Stock solutions of all 9 enzymes were prepared by consecutive dilution of commercially available proteases (Enzo Lifesciences). 3 mL of PBS–dextran (10 mg dextran in 1.0 mL of PBS) are mixed with 75 µL of the nanoplatform dispersion (3.0 mg in 3.0 mL of PBS, see above), 30 µL of inactivated serum, and 30 µL of each of the proteases at every concentration level in PBS. The dispersions were incubated at 25 °C for 60 min, followed by the recording of a fluorescence spectrum at 25 °C using a Fluoromax2 spectrometer (λ_ex_ = 421 nm, λ_em_= 620–680 nm). Inactivation of serum was achieved by heating to 56 °C in an incubator for 45 min, taking the heating time of the serum from RT to the chosen temperature into account, making sure that the serum is heated for a minimum of 30 min. Inactivated serum tested negative with all nine nanoplatforms for protease measurements employed in this study.

## Supporting Information

File 1 Determination of matrix effects on the observed fluorescence intensities of the nanoplatforms, relative error from 10 repetitive protease measurements, and comparison of cancer stages and boxplots for each of the investigated proteases.
