# Supplementary material for: Early breast cancer screening using iron/iron oxide-based nanoplatforms with sub-femtomolar limits of detection
Source: Beilstein J Nanotechnol. 2016 Mar 7;7:364–73. doi: 10.3762/bjnano.7.33 (PMC4901534; doi:10.3762/bjnano.7.33)
Supplement: File 1 — Determination of matrix effects on the observed fluorescence intensities of the nanoplatforms, relative error from 10 repetitive protease measurements, and comparison of cancer stages and boxplots for each of the investigated proteases. [file Beilstein_J_Nanotechnol-07-364-s001.pdf]

Supporting Information

for

Early breast cancer screening using iron/iron oxide-based nanoplatforms with sub-femtomolar limits of detection

Dinusha N. Udukala[‡1], Hongwang Wang[‡1], Sebastian O. Wendel[‡1], Aruni P. Malalasekera[‡1], Thilani N. Samarakoon[1], Asanka S. Yapa[1], Gayani Abayaweera[1], Matthew T. Basel[2], Pamela Maynez[1], Raquel Ortega[1], Yubisela Toledo[1], Leonie Bossmann[1], Colette Robinson[1], Katharine E. Janik[1], Olga B. Koper[1], Ping Li[1], Massoud Motamedi[3], Daniel A. Higgins[1], Gary Gadbury[4], Gaohong Zhu*[5], Deryl L. Troyer*[2] and Stefan H. Bossmann*[1]

Address: [1]Kansas State University, Department of Chemistry, 213 CBC Building, Manhattan, KS, USA, [2]Kansas State University, Department of Anatomy & Physiology, 228 Coles Hall, Manhattan, KS, USA, [3]The University of Texas Medical Branch, 301 University Boulevard, Galveston, TX, USA, [4]Kansas State University, Department of Statistics, 101 Dickens Hall, Manhattan, KS, USA and [5]The First Affiliated Hospital of Kunming Medical University, Department of Nuclear Medicine, 295 Xichang Road, Kunming, Yunnan, PR China

Email: Gaohong Zhu - 1026909611@qq.com; Deryl L. Troyer - troyer@vet.k-state.edu; Stefan H. Bossmann - sbossman@ksu.edu

* Corresponding author

[‡] Equal contributors

Determination of matrix effects on the observed fluorescence intensities of the nanoplatforms, relative error from 10 repetitive protease measurements, and comparison of cancer stages and boxplots for each of the investigated proteases

Page	SI Information
S3	1. Determination of Matrix Effects on the Observed Fluorescence Intensities of the Nanoplatforms (Figures S1, S2)
S4	2. Relative Error from 10 Repetitive Protease Measurements (Figure S3)
S5	3. Comparison of Cancer Stages and Boxplots for Each of the Investigated Proteases (Figures S4, Table S1 (Cathepsin B))
S6	Comparison of Cancer Stages and Boxplots for Each of the Investigated Proteases (Figures S5, Table S2 (Cathepsin L))
S7	Comparison of Cancer Stages and Boxplots for Each of the Investigated Proteases (Figures S6, Table S3 (Urokinase Plasminogen Activator))
S8	Comparison of Cancer Stages and Boxplots for Each of the Investigated Proteases (Figures S7, Table S4 (Matrix Metalloproteinase 1))
S9	Comparison of Cancer Stages and Boxplots for Each of the Investigated Proteases (Figures S8, Table S5 (Matrix Metalloproteinase 2))
S10	Comparison of Cancer Stages and Boxplots for Each of the Investigated Proteases (Figures S9, Table S6 (Matrix Metalloproteinase 3))
S11	Comparison of Cancer Stages and Boxplots for Each of the Investigated Proteases (Figures S10, Table S7 (Matrix Metalloproteinase 7))
S12	Comparison of Cancer Stages and Boxplots for Each of the Investigated Proteases (Figures S11, Table S8 (Matrix Metalloproteinase 9))
S13	Comparison of Cancer Stages and Boxplots for Each of the Investigated Proteases (Figures S12, Table S9 (Matrix Metalloproteinase 13))
S14	Reference

1. Determination of Matrix Effects on the Observed Fluorescence Intensities of the Nanoplatforms

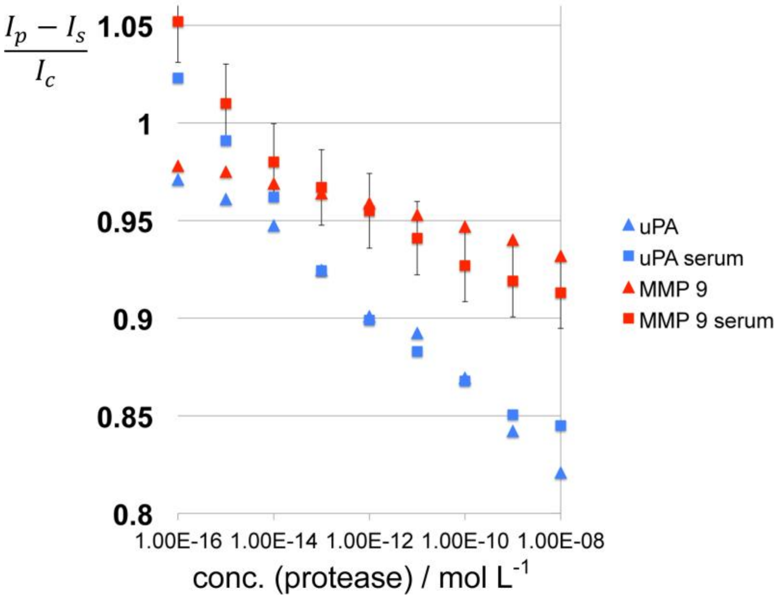

Figure S1: Matrix effects for MMP9 and uPA after 60 min of incubation at 25°C under standard conditions. Triangles: fluorescence readings in PBS; Squares: fluorescence readings in PBS containing inactivated serum. I_p: fluorescence signal after 60 min. of incubation; I_c: fluorescence signal in the absence of protease after 60 min. incubation; I_s: fluorescence signal of serum/PBS-dextran alone. Experimental errors are indicated. A detailed mechanistic discussion of the reasons why uPA and MMP9 are defying the "light switch paradigm" is provided in reference S1.

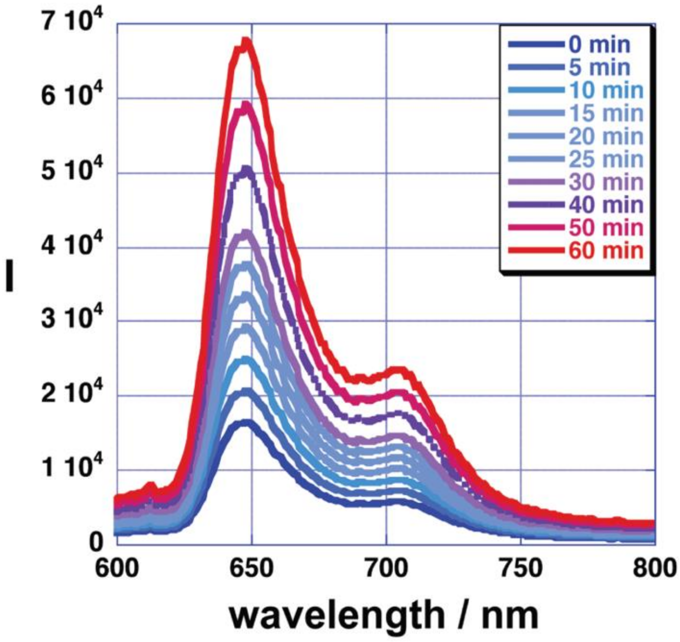

Figure S2: "Light-Switch Effect" of the Fe/Fe_3O_4-nanoplatform for detecting MMP13: fluorescence increase as a function of reaction time under standard conditions at 25°C after addition of 1.0 x 10^{-12} mol l^{-1} of MMP13, with permission from Royal Society of Chemistry (reference S1).

2. Relative Error from 10 Independently Performed Protease Measurements

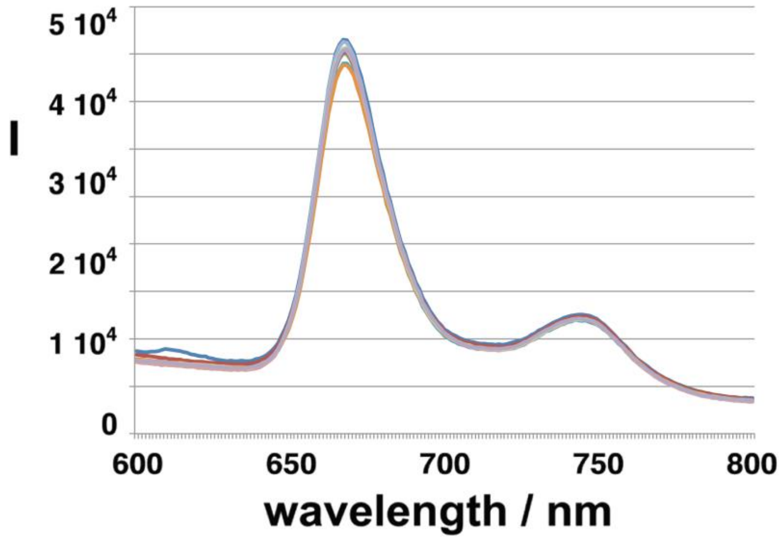

Figure S3: 10 independent repetitions of measuring the activity of the Fe/Fe_3O_4-nanoplatform for detecting MMP13 under standard conditions at 25°C after addition of 1.0 x 10^{-13} mol l^{-1} of MMP13. The relative error was determined to be 3 percent.

3. Boxplots and Bar-Graphs for Nine Proteases (Cathepsin L is also shown in the main text)

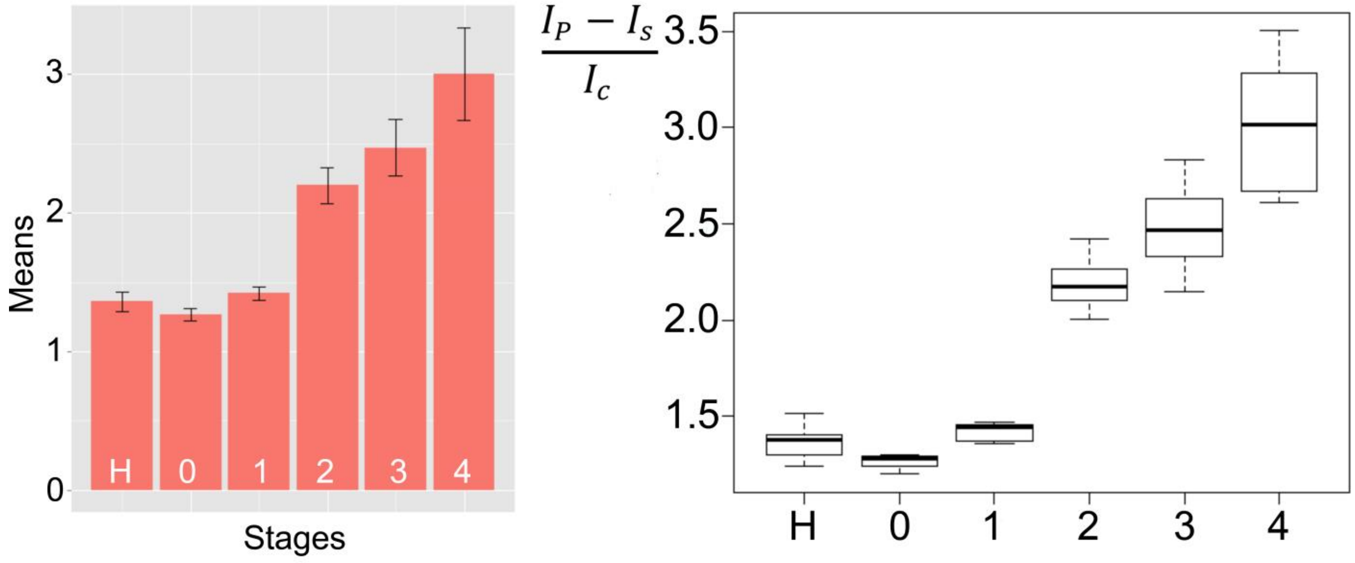

Figure S4: Bar graph (left, showing means and standard deviations) and box plot (right, indicating the observed data range) for cathepsin B. The group sizes are H (apparently healthy control group, n=20), 0: breast cancer stage 0 (n=4), 1: breast cancer stage 1 (n=9), 2: breast cancer stage 2 (n=9), 3: breast cancer stage 3 (n=12); 4: breast cancer stage 4 (n=12). All biospecimens were obtained from the Southeastern Nebraska Cancer Center (SNCC). Breast cancer has been staged according to the TNM staging system.[S1]

Table S1: Means, Standard Deviations, and Average Protease Activities in Serum for Cathepsin B (CTS B).

Stages	Means	Standard Deviation	Average Protease Activity in Serum (mol L^{-1})
H	1.360853	0.07226045	2.4×10^{-16}
0	1.264777	0.04452960	1.4×10^{-16}
1	1.418908	0.04501105	3.3×10^{-16}
2	2.198153	0.12710588	2.6×10^{-14}
3	2.469330	0.20346940	1.1×10^{-13}
4	3.002602	0.33220471	1.8×10^{-12}

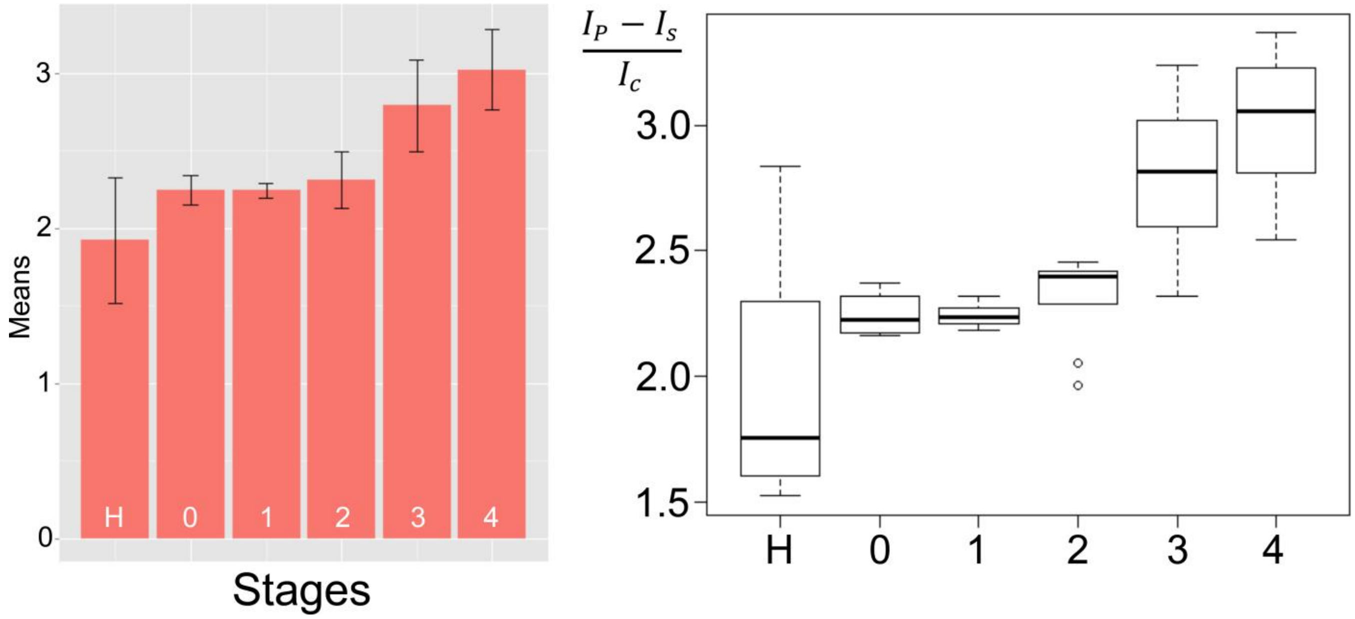

Figure S5: Bar graph (left, showing means and standard deviations) and box plot (right, indicating the observed data range) for cathepsin L. The group sizes are H (apparently healthy control group, n=20), 0: breast cancer stage 0 (n=4), 1: breast cancer stage 1 (n=9), 2: breast cancer stage 2 (n=9), 3: breast cancer stage 3 (n=12); 4: breast cancer stage 4 (n=12). All biospecimens were obtained from the South Eastern Nebraska Cancer Center (SNCC). Breast cancer has been staged according to the TNM staging system.[S1]

Table S2: Means, Standard Deviations, and Average Protease Activities in Serum for Cathepsin L (CTS L).

Stages	Means	Standard Deviation	Average Protease Activity in Serum (mol L^{-1})
H	1.923536	0.40659660	1.8×10^{-15}
0	2.245194	0.09365240	8.5×10^{-15}
1	2.243285	0.04673341	8.4×10^{-15}
2	2.312233	0.18165558	1.1×10^{-14}
3	2.790727	0.29286377	5.4×10^{-14}
4	3.021944	0.25981286	1.2×10^{-13}

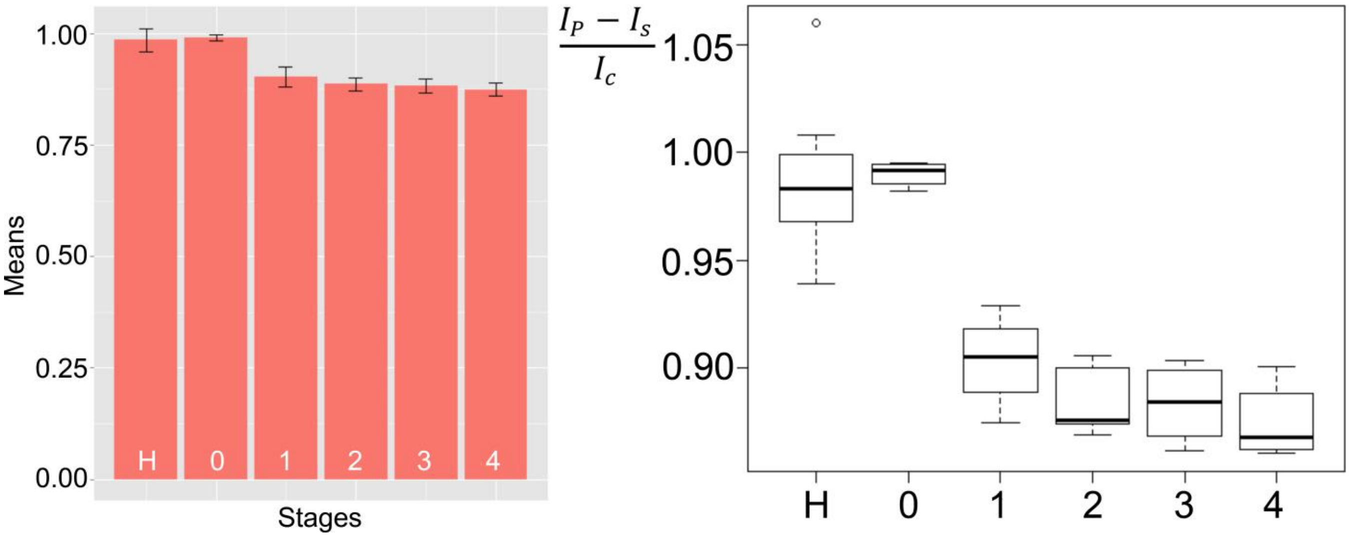

Figure S6: Bar graph (left, showing means and standard deviations) and box plot (right, indicating the observed data range) for urokinase plasminogen activator (uPA). The group sizes are H (apparently healthy control group, n=20), 0: breast cancer stage 0 (n=4), 1: breast cancer stage 1 (n=9), 2: breast cancer stage 2 (n=9), 3: breast cancer stage 3 (n=12); 4: breast cancer stage 4 (n=12). All biospecimens were obtained from the South Eastern Nebraska Cancer Center (SNCC). Breast cancer has been staged according to the TNM staging system.[S1]

Table S3: Means, Standard Deviations, and Average Protease Activities in Serum for Urokinase Plasminogen Activator (uPA).

Stages	Means	Standard Deviation	Average Protease Activity in Serum (mol L^{-1})
H	0.9848335	0.025264087	1.3×10^{-15}
0	0.9900917	0.005991484	7.8×10^{-16}
1	0.9034983	0.022280082	2.1×10^{-12}
2	0.8860111	0.015128888	1.0×10^{-11}
3	0.8832422	0.015965042	1.3×10^{-11}
4	0.8741700	0.014332318	2.0×10^{-11}

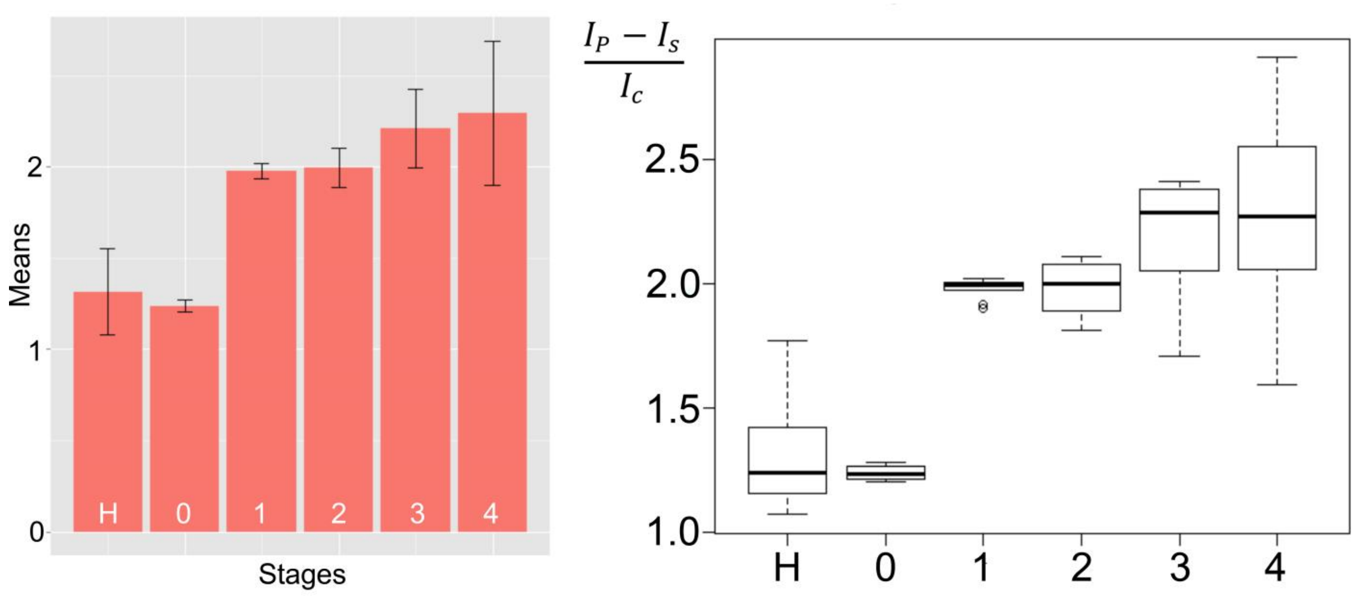

Figure S7: Bar graph (left, showing means and standard deviations) and box plot (right, indicating the observed data range) for matrix metalloproteinase 1 (MMP 1). The group sizes are H (apparently healthy control group, n=20), 0: breast cancer stage 0 (n=4), 1: breast cancer stage 1 (n=9), 2: breast cancer stage 2 (n=9), 3: breast cancer stage 3 (n=12); 4: breast cancer stage 4 (n=12). All biospecimens were obtained from the South Eastern Nebraska Cancer Center (SNCC). Breast cancer has been staged according to the TNM staging system.[S1]

Table S4: Means, Standard Deviations, and Average Protease Activities in Serum for Matrix Metalloproteinase 1 (MMP 1).

Stages	Means	Standard Deviation	Average Protease Activity in Serum (mol L^{-1})
H	1.314616	0.23507424	1.6×10^{-15}
0	1.236894	0.03339194	5.9 x 10-^{16}
1	1.978331	0.04220099	7.8×10^{-12}
2	1.995024	0.10799028	9.6×10^{-12}
3	2.210357	0.21628141	1.5×10^{-10}
4	2.292405	0.39496657	4.3×10^{-10}

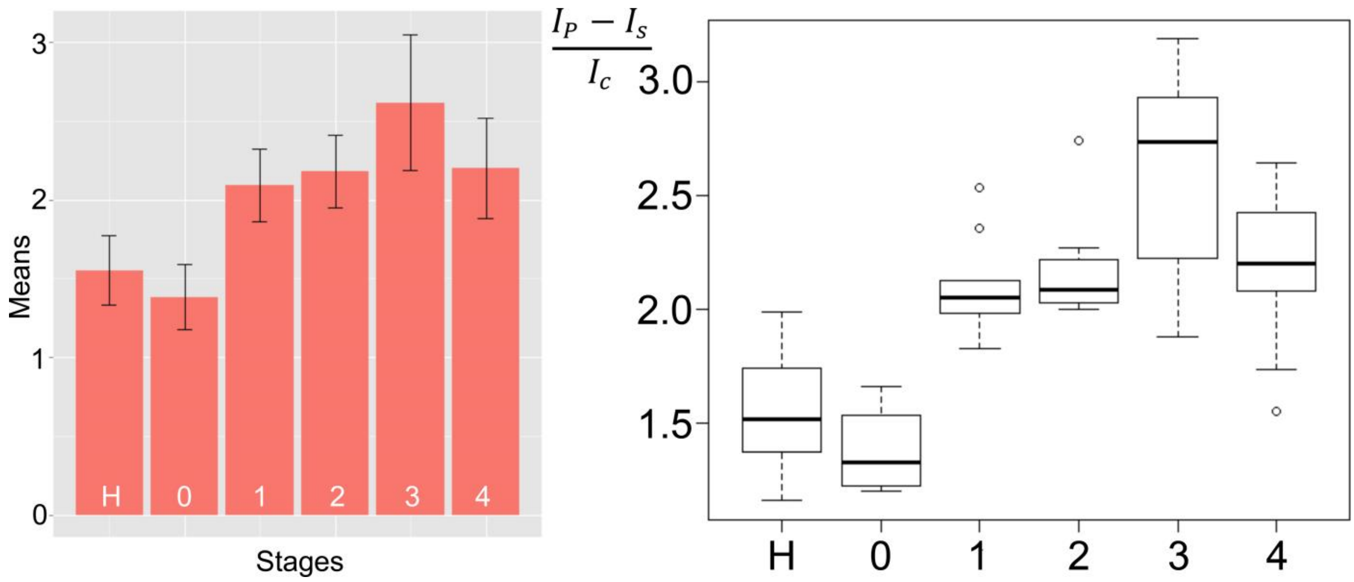

Figure S8: Bar graph (left, showing means and standard deviations) and box plot (right, indicating the observed data range) for matrix metalloproteinase 2 (MMP 2). The group sizes are H (apparently healthy control group, n=20), 0: breast cancer stage 0 (n=4), 1: breast cancer stage 1 (n=9), 2: breast cancer stage 2 (n=9), 3: breast cancer stage 3 (n=12); 4: breast cancer stage 4 (n=12). All biospecimens were obtained from the South Eastern Nebraska Cancer Center (SNCC). Breast cancer has been staged according to the TNM staging system.[S1]

Table S5: Means, Standard Deviations, and Average Protease Activities in Serum for Matrix Metalloproteinase 2 (MMP 2)

Stages	Means	Standard Deviation	Average Protease Activity in Serum (mol L^{-1})
H	1.551050	0.2210302	4.0×10^{-15}
0	1.381780	0.2078706	6.4×10^{-16}
1	2.093351	0.2287855	1.4×10^{-12}
2	2.181590	0.2301482	3.6×10^{-12}
3	2.616193	0.4300199	4.0×10^{-10}
4	2.200369	0.3192078	4.4×10^{-12}

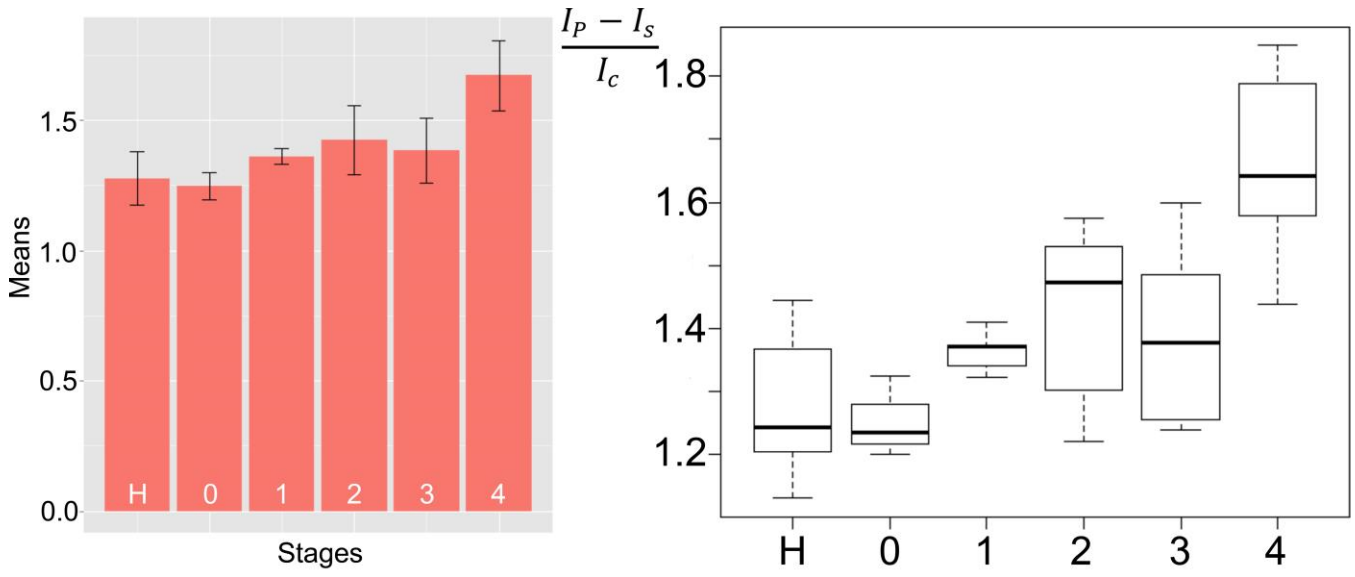

Figure S9: Bar graph (left, showing means and standard deviations) and box plot (right, indicating the observed data range) for matrix metalloproteinase 3 (MMP 3). The group sizes are H (apparently healthy control group, n=20), 0: breast cancer stage 0 (n=4), 1: breast cancer stage 1 (n=9), 2: breast cancer stage 2 (n=9), 3: breast cancer stage 3 (n=12); 4: breast cancer stage 4 (n=12). All biospecimens were obtained from the South Eastern Nebraska Cancer Center (SNCC). Breast cancer has been staged according to the TNM staging system.[S1]

Table S6: Means, Standard Deviations, and Average Protease Activities in Serum for Matrix Metalloproteinase 3 (MMP 3)

Stages	Means	Standard Deviation	Average Protease Activity in Serum (mol L^{-1})
H	1.276774	0.10293781	3.4×10^{-16}
0	1.248445	0.05253079	2.2×10^{-16}
1	1.361347	0.02882905	1.2×10^{-15}
2	1.423178	0.13355403	2.9×10^{-15}
3	1.383381	0.12401589	1.6×10^{-15}
4	1.670680	0.13369459	1.2×10^{-13}

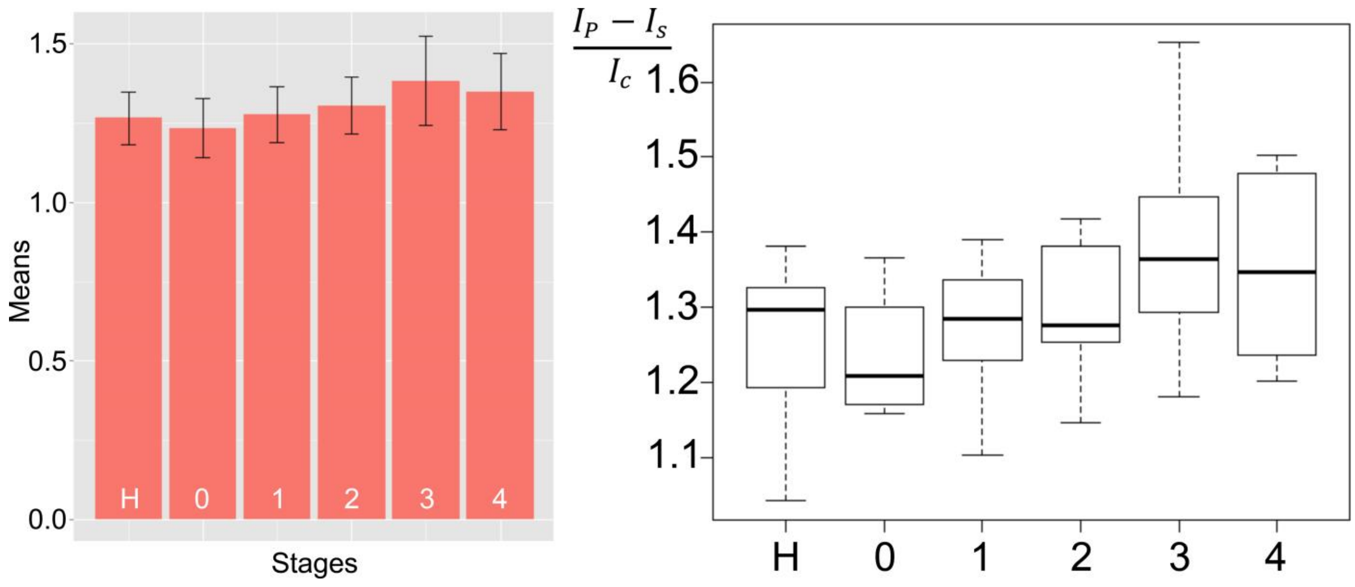

Figure S10: Bar graph (left, showing means and standard deviations) and box plot (right, indicating the observed data range) for matrix metalloproteinase 7 (MMP 7). The group sizes are H (apparently healthy control group, n=20), 0: breast cancer stage 0 (n=4), 1: breast cancer stage 1 (n=9), 2: breast cancer stage 2 (n=9), 3: breast cancer stage 3 (n=12); 4: breast cancer stage 4 (n=12). All biospecimens were obtained from the South Eastern Nebraska Cancer Center (SNCC). Breast cancer has been staged according to the TNM staging system.[S1]

Table S7: Means, Standard Deviations, and Average Protease Activities in Serum for Matrix Metalloproteinase 7 (MMP 7).

Stages	Means	Standard Deviation	Average Protease Activity in Serum (mol L^{-1})
H	1.265858	0.08413963	6.4×10^{-16}
0	1.235173	0.09279042	4.5×10^{-16}
1	1.276960	0.08834555	7.2×10^{-16}
2	1.306292	0.09032285	1.0×10^{-15}
3	1.384025	0.14060255	2.5×10^{-15}
4	1.349619	0.12004177	1.7×10^{-15}

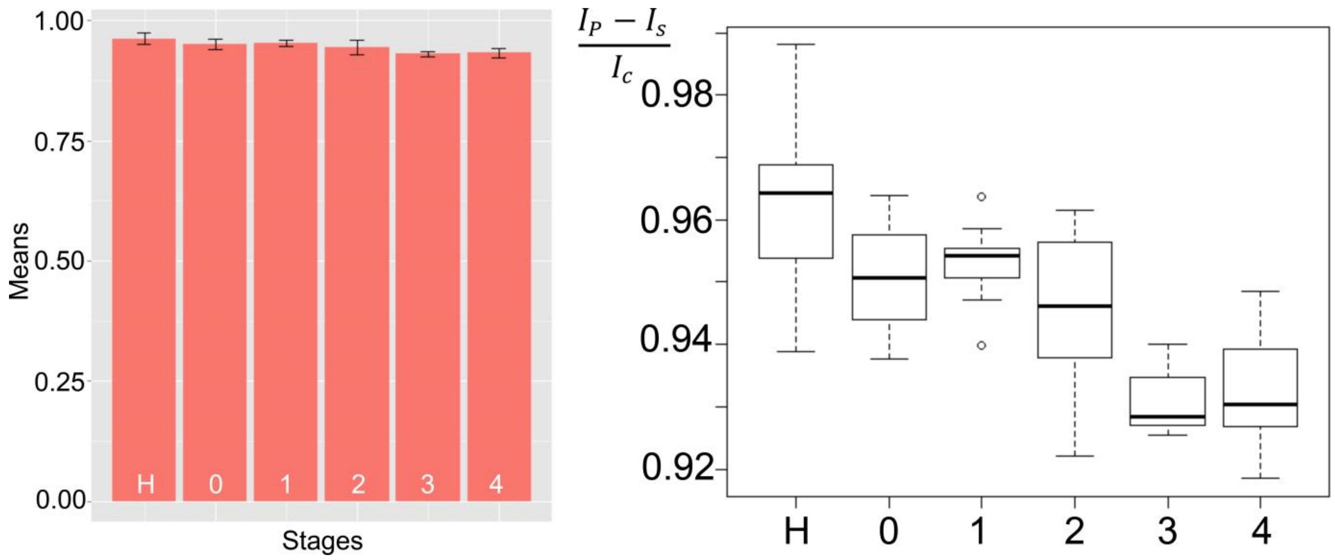

Figure S11: Bar graph (left, showing means and standard deviations) and box plot (right, indicating the observed data range) for matrix metalloproteinase 9 (MMP 9). The group sizes are H (apparently healthy control group, n=20), 0: breast cancer stage 0 (n=4), 1: breast cancer stage 1 (n=9), 2: breast cancer stage 2 (n=9), 3: breast cancer stage 3 (n=12); 4: breast cancer stage 4 (n=12). All biospecimens were obtained from the South Eastern Nebraska Cancer Center (SNCC). Breast cancer has been staged according to the TNM staging system.[S1]

Table S8: Means, Standard Deviations, and Average Protease Activities in Serum for Matrix Metalloproteinase 9 (MMP 9).

Stages	Means	Standard Deviation	Average Protease Activity in Serum (mol L^{-1})
H	0.9622952	0.011980785	8.6×10^{-13}
0	0.9507300	0.010709674	5.9×10^{-12}
1	0.9530756	0.006783337	4.0×10^{-12}
2	0.9439189	0.014798504	1.8×10^{-11}
3	0.9308592	0.005204805	1.6×10^{-10}
4	0.9327775	0.009121815	1.2×10^{-10}

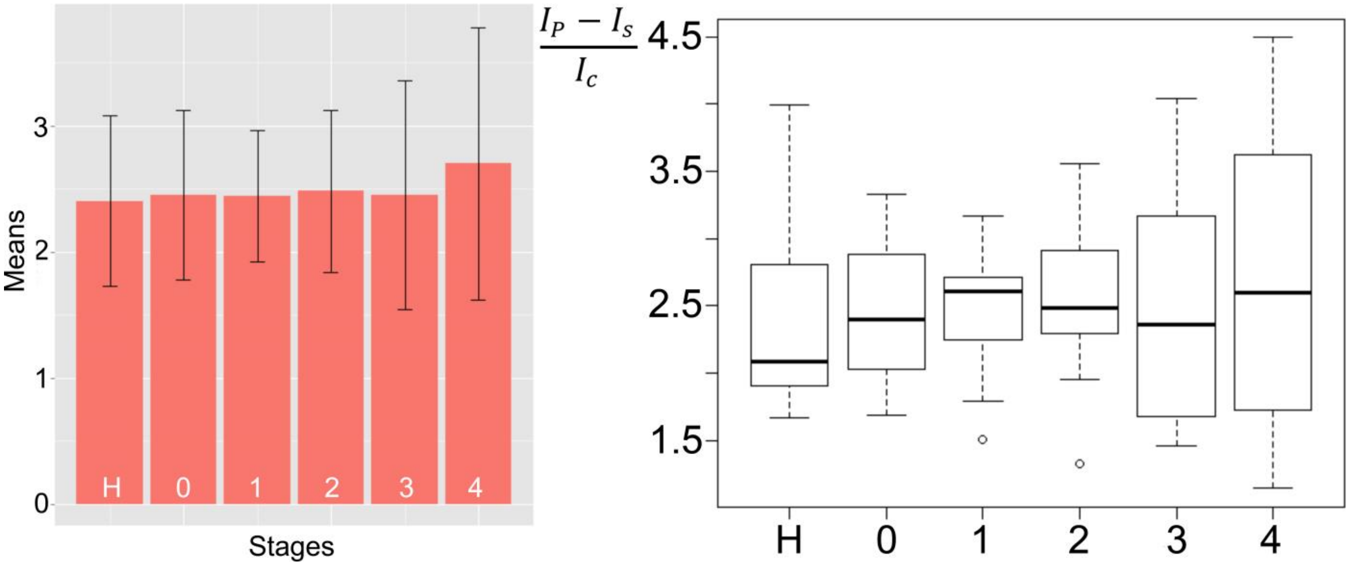

Figure S12: Bar graph (left, showing means and standard deviations) and box plot (right, indicating the observed data range) for matrix metalloproteinase 13 (MMP 13). The group sizes are H (apparently healthy control group, n=20), 0: breast cancer stage 0 (n=4), 1: breast cancer stage 1 (n=9), 2: breast cancer stage 2 (n=9), 3: breast cancer stage 3 (n=12); 4: breast cancer stage 4 (n=12). All biospecimens were obtained from the South Eastern Nebraska Cancer Center (SNCC). Breast cancer has been staged according to the TNM staging system.[S1]

Table S9: Means, Standard Deviations, and Average Protease Activities in Serum for Matrix Metalloproteinase 13 (MMP 13).

Stages	Means	Standard Deviation	Average Protease Activity in Serum (mol L^{-1})
H	2.402871	0.6767673	3.2×10^{-15}
0	2.454853	0.6726423	3.7×10^{-15}
1	2.447914	0.5209525	3.6×10^{-15}
2	2.483194	0.6442519	4.0×10^{-15}
3	2.453076	0.9055961	3.7×10^{-15}
4	2.701158	1.0781773	7.5×10^{-15}

S1: Singletary, S.E., Connolly, J.L., 2006. Breast cancer staging: working with the sixth edition of the AJCC Cancer Staging Manual. CA Cancer J Clin 56(1), 37-47; quiz 50-31.
